# Establishment of an RPA-CRISPR/Cas12a combined diagnostic system for *Pneumocystis jirovecii* pneumonia

**DOI:** 10.1371/journal.pntd.0012922

**Published:** 2025-03-18

**Authors:** Yun Wu, Yuhan Shao, Wei Li, Ying Yu, Xia Rao, Jingyi Li, Nicholas R. Waterfield, Guowei Yang

**Affiliations:** 1 Beijing Institute of Tropical Medicine, Beijing Friendship Hospital, Capital Medical University, Beijing, China; 2 Warwick Medical School, Warwick University, Coventry, United Kingdom; University of Florida, UNITED STATES OF AMERICA

## Abstract

*Pneumocystis jirovecii* causes severe pneumonia in immunocompromised individuals, leading to high mortality and an economic burden. There is a need for early detection methods suitable for low-resource settings and rapid point-of-care diagnostics. This study developed a detection method using Recombinase Polymerase Amplification (RPA) followed by CRISPR/Cas12a with fluorescence detection. The RPA primers and CRISPR-derived RNAs (crRNAs) were specifically designed to target the mitochondrial small subunit rRNA (mtSSU rRNA) gene of *P. jirovecii*. A total of 83 clinical samples were tested using this method, including 39 confirmed and 44 suspected cases of *P. jirovecii* infection. The combination of crRNA5 and crRNA6 demonstrated higher sensitivity compared to the current real-time PCR detection method, with a limit of detection (LOD) of 1 copy per reaction and showed no cross-reactions with other respiratory pathogens. The concordance of this method was validated with both infected and non-infected patients. In conclusion, the method developed in this study potentially provides a highly sensitive and rapid tool suitable for the early and on-site detection of *P. jirovecii* pneumonia. Furthermore, this method holds potential applications for the detection of other human pathogens, representing a significant advancement in diagnostic capabilities for low-resource settings.

## Author summary

*Pneumocystis jirovecii* pneumonia (PJP) is known to exhibit a high fatality rate in immunocompromised population, and prophylactic medication has been proven effective. However, the current diagnostic methods, such as microscopic and PCR examination, are impractical to be used in primary healthcare settings for more rapid and cost-effective diagnosis and impact the effectiveness of treatment strategies. Here, utilizing Recombinase Polymerase Amplification (RPA) coupled with CRISPR/Cas12a fluorescence detection, an innovative diagnostic approach was developed and evaluated with 83 clinical samples. Considering the detection performance of this new method, it provided a reliable point-of-care solution for PJP. Further, it implied that the combination of RPA and CRISPR/Cas12a holds significant promise for advancing molecular diagnostics in both clinical and field-based settings.

## Introduction

*Pneumocystis jirovecii* is a fungal pathogen that causes severe pneumonia in immunocompromised individuals, such as those with HIV/AIDS, organ transplant recipients, autoimmune, inflammatory disease patients and those undergoing chemotherapy [1,2]. The high mortality rate and significant economic burden associated with *P. jirovecii* pneumonia (PJP) necessitate the development of early diagnostic methods to reduce mortality and healthcare costs [3].

Currently, the diagnosis of PJP relies on microscopic examination and PCR testing of respiratory specimens. However, the low sensitivity of microscopy examination may prolong the diagnosis process, delaying treatment [4]. The PCR test requires specialized laboratory facilities, making it impractical to be used in primary healthcare settings. Thus, it presents a critical gap in the early and rapid diagnosis of PJP, particularly in resource-limited areas. Therefore, there is a pressing need for more accurate, rapid, and cost-effective diagnostic tools to detect *P. jirovecii.*

Recombinase Polymerase Amplification (RPA) is an isothermal nucleic acid amplification method that operates efficiently at a constant temperature, typically between 37- 42°C. RPA leverages the activity of recombinase enzymes, which facilitate the binding of primers to the target DNA and a strand-displacing DNA polymerase to synthesize new DNA strands [5]. The isothermal nature of RPA makes it suitable for low-resource settings and rapid point-of-care diagnostics without the complex thermal cycling equipment. RPA can amplify nucleic acids in as little as 20 minutes, which is significantly faster than traditional PCR methods and makes it ideal for time-sensitive diagnostics [6]. Initially identified as a bacterial adaptive immune mechanism, the CRISPR/Cas system has been repurposed for various molecular diagnostic applications due to its high specificity and programmability [7]. Among these various CRISPR systems, CRISPR/Cas12a stands out in diagnostic settings because of its collateral cleavage activity, which can be utilized to generate a detectable signal upon target recognition. Further, different from CRISPR/Cas13a which operates on RNA, CRISPR/Cas12a can work on DNA. This system has previously been applied to successfully detect various pathogens, including viruses and bacteria [8].

To improve upon the current PJP diagnosis methods, this study explored the potential utilization of RPA combined with CRISPR/Cas12 technology for the rapid and cost-effective detection of *P. jirovecii*. The mitochondrial small subunit rRNA (mtSSU rRNA) gene is conserved across various *P. jirovecii* strains with numerous copies, providing an optimal target for precise detection [9,10]. Targeted primers and several CRISPR-derived RNAs (crRNAs) were designed aimed at *P. jirovecii* mtSSU rRNA. Furthermore, the use of multiple crRNAs here not only enhances the test’s sensitivity but also minimizes the potential of producing false negatives. A comprehensive validation of this test was conducted using 83 clinical samples from patients with confirmed and suspected *P. jirovecii* pneumonia. The results demonstrated that this new method could reliably identify *P. jirovecii* with high sensitivity and specificity. Overall, this method not only provides a powerful tool for reliable, rapid, and cost-effective solutions to *P. jirovecii* pneumonia diagnosis but also demonstrates that the combination of RPA and CRISPR/Cas12a holds significant promise for advancing molecular diagnostics for other pathogens in both clinical and field-based settings.

## Materials and methods

### Ethics statement

The project has been approved by the Ethics Committee of Beijing Friendship Hospital (Beijing, China) with approval number of 2024-P2-323-01 and that all clinical samples were obtained from an existing sample collection. All samples were anonymized.

### Patients and samples

In this study, a total of 83 sputum and BALF samples were collected from patients at Beijing Friendship Hospital, Capital Medical University, from Sep 2019 to Feb 2023. The clinical diagnostic criteria for *P. jirovecii* pneumonia were based on the clinical manifestation, imaging characteristics and conventional PCR amplification of the *P. jirovecii* mitochondrial large subunit ribosomal RNA (mtLSU rRNA) gene from sputum and bronchoalveolar lavage fluid (BALF) specimens [11]. Among these samples, 39 samples (25 sputum and 14 BALF) were from patients clinically diagnosed with *P. jirovecii* pneumonia (median age 65 years, ranging from 24 to 84; 28males and 11females). We confirmed the diagnosis by real-time PCR amplification of the *P. jirovecii* mitochondrial small subunit ribosomal RNA (mtSSU rRNA) gene. The remaining 44 samples (36 sputum and 8 BALF) were obtained from patients, who were clinically suspected to have *P. jirovecii* pneumonia (median age 66years, ranging from 20 to 98; 30males and 14 females). However, these samples were negative in conventional PCR (targeting the mtLSU rRNA gene) and real-time PCR (targeting the mtSSU rRNA gene). As a result of the follow-up clinical evaluations, we ruled them out for infection or colonization.

### DNA extraction from clinical samples

DNA was extracted from fresh sputum or BALF samples obtained from patients. The samples were pre-treated with 1N NaOH (1:1 v/v for sputum and 2:1 v/v for BALF) at 60 °C for 1 hour to ensure complete liquefaction before centrifugation at 8000 rpm for 5 minutes. The pellet was washed twice with saline. DNA were extracted from the pellet using a kit (TIANGEN, DP305, Beijing, CHN) according to the manufacturer’s instructions and stored at −80 °C for further analysis.

### Design of primers and crRNAs

RPA primers were designed using Beacon Designer (v8.00, PREMIER Biosoft, Palo Alto, CA, USA) and crRNAs were designed using Online tools (http://crispor.tefor.net) based on the conserved region of mtSSU rRNA gene sequences of *P. jirovecii.* The specificity was evaluated using Primer Blast of NCBI. All sequences were synthesized by Sangon Biotech Co., Ltd (S1 and S2 Tables). crRNAs were aliquoted and stored at −80 °C until use.

### The crRNAs screening

The amplified product from patients with *P. jirovecii* pneumonia were served as the template for the crRNAs test. The reaction contained 2 µl of 10× buffer (Bio-lifesci, M20301-0500, Guangzhou, China), 100 nM crRNA, 500 nM fluorescence probe (Bio-lifesci, AX104121254, Guangzhou, China), 100 nM Cas12a, 1 µl template, and supplemented with dH_2_O to achieve a total volume of 20 µl. The reaction was carried out at 45 °C for 30 minutes with fluorescence readings taken every minute.

### Target DNA amplification using RPA

In this study, the mtSSU rRNA gene of *P. jirovecii* was used as the target DNA. Besides the manufacturer’s instructions, we also conducted systematic optimization to establish the reaction system as previously described [12]. The target fragment was enriched with RPA nucleic acid amplification reagent (Freeze-dried granules) (HuicH Biotech, HP80201, Shanghai, China), 400 nM of primer PJ-RPA-7F and PJ-RPA-7R, template DNA (5–50 ng) and add ddH_2_O to 25 μl, followed by the addition of magnesium acetate (Freeze-dried granules) (HuicH Biotech, HP8020, Shanghai, China). The reaction was carried out at 42 °C for 15-30 min. Confirmed by PCR amplification and DNA sequencing, The plasmid mtSSU/pUC19 was employed as the positive control, while ddH_2_O was utilized as the negative control in this study.

### CRISPER/Cas12a detection with fluorescence probe

The reaction conditions was optimized as previously described [13]. The total volume of the CRISPER/Cas12a reaction system was 20 μl, including 2 μl 10×buffer (Bio-lifesci, M20301-0500, Guangzhou, China), 100 nΜ Cas12a (Bio-lifesci, M20301-0500, Guangzhou, China), 50 nΜ crRNA5, 50 nΜ crRNA6, 500 nM fluorescence probe (Bio-lifesci, AX104121254, Guangzhou, China), 1 μl template (1:100 dilution of RPA product), and add ddH_2_O to 20 μl. The reaction was carried out at 45 °C for 30 minutes with fluorescence readings taken every minute.

### Analytical sensitivity and specificity

The limit of detection (LOD) was defined as the lowest number of detectable copies based on repeated tests (n = 3), using 100, 50, 25, 12, 6, 3 and 1 plasmid copies/reaction. The specificity was evaluated using DNA extracted from cultured *Aspergillus fumigatus*, *Aspergillus flavus*, and *Aspergillus niger*, as well as patients infected with *Mycobacterium tuberculosis, Klebsiella pneumoniae, Pseudomonas aeruginosa,* Influenza virus*,* Cytomegalovirus*,* Epstein-Barr virus*, Escherichia coli,* Group A Streptococcus*, Neisseria,* Hemolytic Staphylococcus*, Candida albicans, Candida glabrata, Enterobacter cloacae* and *Candida tropicalis*.

### Detection of clinical samples using qPCR

The previous well-established qPCR method was employed to evaluate clinical samples [10]. The qPCR was executed in a 20 μl reaction comprising 300 nm of primer PJ-F and PJ-R, 200nm PJ-probe (5’-FAM/3’-BHQ1) for *P. jirovecii*, 10 μl GoTaq Probe qPCR Master Mix (Promega, A6101, Madison, WI, USA), and 1 μl template DNA (5–50 ng). The reaction took place in the Applied Biosystems 7500 Fast Real-Time PCR System (ABI) with an initial phase at 95 °C for 2 minutes, followed by 40 cycles of 95 °C for 15 seconds and 58 °C for 50 seconds. Each sample was assessed in duplicates, with plasmid mtSSU/pUC19 as the positive control and ddH_2_O as the negative control in all experiments.

### Clinical sensitivity and specificity

A total of 83 clinical samples were utilized to validate the performance of this new assay. DNA extraction and RPA-CRISPR/Cas12a based detection system were conducted following the prescribed procedure, and the detection outcomes were compared with the clinical diagnoses and qPCR test results.

### Statistical analysis

Statistical analysis was conducted using SPSS software version 20.0 and visualized with GraphPad Prism version 9.0. Repeated measurements were represented using the mean ± standard deviation, unpaired 2-tailed Student’s t tests were used to analyze the differences statistically compare between groups. *P* < 0.05 was considered as statistical significance. The qPCR data were expressed as medians (ranges).

## Results

### Screening and sensitivity of crRNAs for *P. jirovecii*

**Fig 1:**
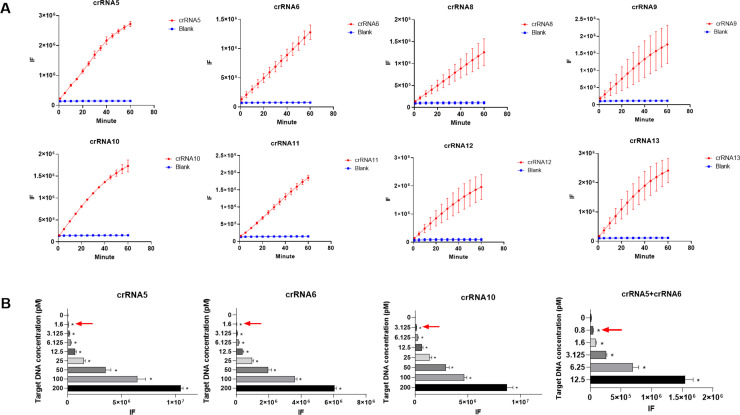
Screening of crRNAs for RPA-CRISPR/Cas12a combined diagnostic system for *P. jirovecii.* **(A)** Eight candidate crRNAs testing, the amplification products of positive patients served as templates, and ddH_2_O as the negative control. **(B)** Evaluation the detection sensitivity of crRNA5, crRNA6, crRNA10, and crRNA5+crRNA6 using mtSSU/pUC19 plasmid at different concentrations. ddH_2_O as the negative control. * *P*<0.05 and the red arrow showed the detection sensitivity of different crRNAs.

Consistent with the *P. jirovecii* mtSSU rRNA gene sequence (GeneBank Accession No. JX499143), ten candidate crRNAs were devised which showed strong specificity for the target gene (S1 Table). Experimental validation confirmed that 8 of these 10 crRNAs could bound to their target and cleave the single-stranded DNA probe to release fluorescent signal effectively. We then diluted the amplification products from patient samples to 1:10, 1:100, 1:1000, and 1:10000, which served as templates for detecting the sensitivity of the rest eight crRNAs. The results indicated that crRNA5, crRNA6, and crRNA10 were capable of detecting template concentrations as low as 1:1000, exhibiting superior performance compared to other crRNAs tested here (Fig 1A). To compare the detection sensitivity of these three crRNAs, we further performed sequential dilutions of the template, ranging from 200 pM to 0.8 pM. It showed that, while crRNA10 exhibited a sensitivity of 3.125 pM, crRNA5 and crRNA6 possessed detection sensitivities of 1.6 pM. We then combined crRNA5 and crRNA6 for further test, which revealed improved detection sensitivity compared to crRNA5 or crRNA6 alone (Fig 1B). Thus, our results showed that the combination of crRNA5 and crRNA6 is best suited for the *P. jirovecii* detection assay.

### Detection of *P. jirovecii* using the RPA-CRISPR/Cas12a combined detection assay

**Fig 2:**
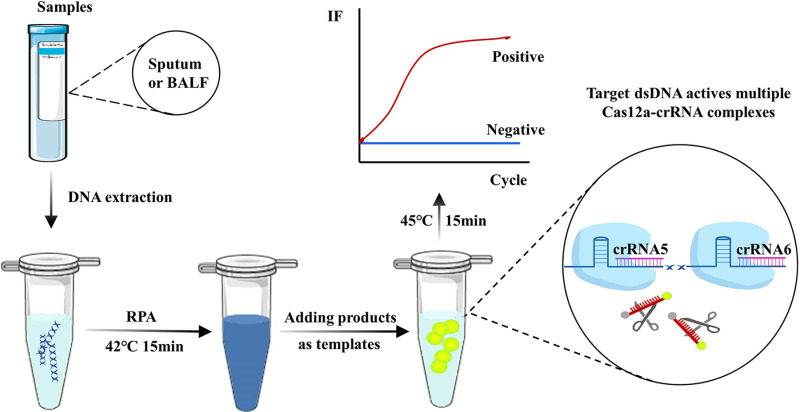
The workflow of RPA-CRISPR/Cas12a detection system for *P. jirovecii.* This workflow includes extracting DNA from sputum or BALF, using it as a template in the RPA reaction, adding the amplified product to the CRISPR/Cas12a detection system, and detecting the fluorescence signal every minute. Some materials are from Smart Servier Medical Art (https://smart.servier.com/), used under the CC BY 4.0 license.

**Fig 3:**
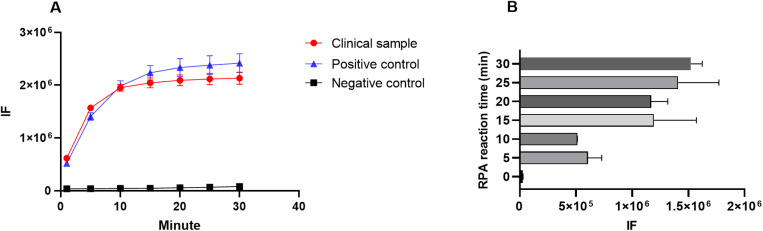
Establishment of an RPA-CRISPR/cas12a combined assay for *P. jirovecii* detection. **(A)**: Test clinical sample using the new assay. The mtSSU/pUC19 plasmid as positive control and ddH_2_O as negative control. **(B)**: The correlation between the fluorescence signal intensity and the extension of RPA reaction time for the *P. jirovecii* detection.

To establish the RPA-CRISPR/Cas12a combined *P. jirovecii* detection assay, RPA primers PJ-RPA-7F and PJ-RPA-7R were designed in conjunction with crRNA5+crRNA6 (S2 Table). The flowchart illustrates the process used in the new method. It includes three main steps: DNA sample extraction, RPA amplification, and CRISPR/Cas12a detection of fluorescence signals (Fig 2). A clinical sample was tested using this new assay in accordance with this workflow. A *P. jirovecii* mtSSU plasmid (1 × 10^9^copies/reaction) was used as a positive control and ddH_2_O as a negative control. The results indicated that both clinical samples and positive controls detected the released fluorescent signals, but the negative control did not (Fig 3A). Further, we tested the fluorescence signal intensity with variable RPA reaction times. Although the reaction time of 5–30 minutes produced high fluorescence signals, notably, the signal for 15–30 minutes gave better effective differentiation (Fig 3B). Our result demonstrated that the new RPA-CRISPR/Cas12a based assay developed here exhibits a good potential application for the detection of *P. jirovecii*.

### Assessing the analytical sensitivity and specificity of the RPA-CRISPR/Cas12 combined detection method

**Fig 4:**
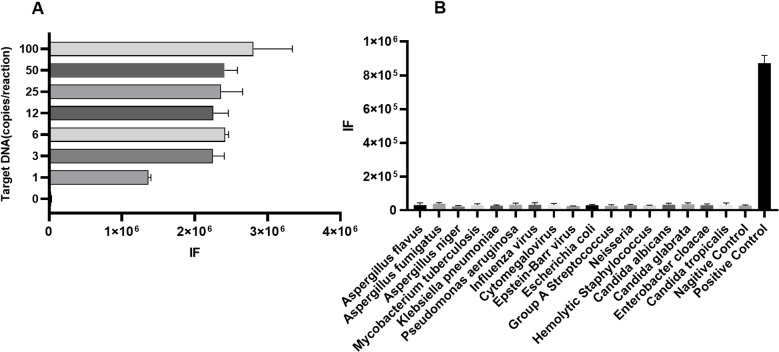
Evaluation of sensitivity and specificity of the new method. **(A)**: Sensitivity evaluation is performed with a series concentration of mtSSU/pUC19 plasmid. **(B)**: Specificity is evaluated with DNA of *A. fumigatus*, *A. flavus*, *A. niger*, *M. tuberculosis*, *K. pneumoniae*, *P. aeruginosa*, Influenza virus, Cytomegalovirus, Epstein-Barr virus, *E. coli*, Group A Streptococcus, *Neisseria*, Hemolytic Staphylococcus, *C. albicans*, *C. glabrata*, *E. cloacae*, and *C. tropicalis*. The positive control is mtSSU/pUC19 plasmid and ddH_2_O as the negative control.

To evaluate the analytical performance of this new assay, the limit of detection (LOD) was examined. It revealed that it is capable of detecting mtSSU/pUC19 plasmids at levels as low as 1 copy/ reaction (Fig 4A). Importantly, the primers used in the assay did not recognize DNA from samples of *A. fumigatus*, *A. flavus*, *A. niger*, *M. tuberculosis*, *K. pneumoniae*, *P. aeruginosa*, Influenza virus, Cytomegalovirus, Epstein-Barr virus, *E. coli*, Group A Streptococcus, *Neisseria*, Hemolytic Staphylococcus, *C. albicans*, *C. glabrata*, *E. cloacae*, and *C. tropicalis* (Fig 4B). Our results showed that this new assay might be suitable for *P. jirovecii* detection with high sensitivity and specificity.

### Evaluation of the diagnostic performance of the assay using clinical samples

A total of 83 clinical samples were tested, including 39 confirmed and 44 suspected *P. jirovecii* infection cases. These samples were verified using a previously established qPCR method for *P. jirovecii* [10]. The burden of *P. jirovecii* was quantified at 1.1 × 10^6^ (6.34 × 10^3^, 2.8 × 10^9^) copies by this method, and the results are consistent with clinical diagnoses (S3 Table). These 83 clinical samples were then tested using our new method based on RPA-CRISPR/Cas12a. The clinical validation confirmed that the new method achieved complete concordance with patients infected with *P. jirovecii* as well as those who were not infected (Table 1).

**Table 1:** Clinical sensitivity and specificity of the new assay for diagnosing *P. jirovecii* infection.

Type	Sample type	Diagnosed clinical case		The new assay		Agreement rate%
		+	–	+	–	
*P. jirovecii* infection (n=39)	Sputum/BALF	39	0	39	0	100% (39/39)
Non-*P. jirovecii* infection (n=44)	Sputum/BALF	0	44	0	44	100% (44/44)
Total (n=83)	Sputum/BALF	39	44	39	44	100%

*P. jirovecii*: *Pneumocystis jirovecii*; BALF: bronchial alveolar lavage fluid.

Our evaluations revealed that both the new method described in this study and the previously established real-time PCR method exhibit high sensitivity and specificity to detect the mtSSU rRNA gene of *P. jirovecii*. However, the qPCR method takes 72 minutes and requires a specialized laboratory with Real-time PCR instrument, while this new method only takes 45 minutes and requires only a water bath and a portable fluorescence measurement device. The new method proves to be not only significantly faster but also more cost-effective and convenient than real-time PCR. All these features make this new method suitable for on-site testing and early diagnosis of *P. jirovecii* infections, which would reduce medical costs.

## Discussion

In this study, we developed and validated a novel detection method utilizing both the RPA and CRISPR/Cas12a technology for identifying *P. jirovecii*. This method demonstrated high sensitivity and specificity for the detection of the mtSSU rRNA gene of *P. jirovecii* in clinical samples. Our study exhibits the excellent potential for application of CRISPR/Cas12a as a powerful diagnostic tool for *P. jirovecii* pneumonia and provides a rapid, accurate, and cost-effective alternative to existing diagnostic methods.

This study introduces a novel RPA-CRISPR/Cas12-based detection method for *P. jirovecii*. Multiple crRNA binding sites enhance Cas12a activation, resulting in greater cleavage activity [8,14,15]. Two crRNAs were designed for this method, which bind to different sites on the amplified fragment. Our further experiments confirmed that, compared to a single crRNA, two crRNAs exhibited higher sensitivity (Fig 1B). The application of two crRNAs also effectively minimizes off-target effects and prevents false negatives to ensure the accuracy. Compared to the sensitivity of the previously reported method using CRISPR/Cas13a (2 copies for detecting *P. jirovecii*) [16], a LOD of 1 copy/reaction for this method was indicated in the performance evaluations. The ability to detect *P. jirovecii* at very low copy numbers (1 copy/reaction) suggests that this method could be particularly useful in screening high-risk populations, such as HIV-positive patients or those undergoing immunosuppressive therapy, who may harbor low levels of the pathogen without exhibiting overt symptoms [1,17]. We validated this method with several additional respiratory pathogens affecting immunocompromised patients, and demonstrated good specificity (Fig 4B). As the DNA samples of *Coccidioides* species are unavailable, an in-silico analysis was employed instead, which indicated no theoretical cross-reactivity. It should be noted that due to the limited sample size here, this study may not cover all co-infection scenarios. Future research will include multicenter studies in order to expand the sample size and include more co-infecting respiratory pathogens. Moreover, the previously reported real-time PCR method, which targets the same marker-mtSSU rRNA of *P. jirovecii*, achieved 100% concordance with both confirmed *P. jirovecii*-infected patients and non-infected individuals [10]. In this study, clinical validation demonstrated that this method showed a similar performance as the previous one. We expect that many factors might influence the interpretation of this assay, such as host-related (immunosuppression, pulmonary comorbidities, antimicrobial treatment), sample-related (collection variability, contamination, quality), technical (cross-reactivity, extraction efficiency, PCR inhibition), and environmental (seasonal variation, geographic strain differences, antifungal exposure). Considering these potential confounding factors, further investigations are recommended to optimize the performance and quality control of the method described here. Indeed, the diagnosis of *P. jirovecii* requires a comprehensive integration of molecular diagnostics and clinical evaluations. The CRISPR/Cas12a system’s rapid turnaround time and relatively straightforward protocol make it suitable for point-of-care testing, which could be integrated into routine clinical workflows to enhance diagnostic efficiency, ultimately contributing to better patient care and management [18,19]. Thus, our results implied that CRISPR/Cas12a would be a good alternative for *P. jirovecii* screening with potential clinical implications*.*

Timely and accurate detection of *P. jirovecii* can lead to earlier intervention using appropriate antifungal therapy, potentially reducing morbidity and mortality associated with delayed treatment [20]. Although the new method described here cannot accurately quantify and differentiate colonization from infection, it does reduce detection time to 45 minutes, compared to 72 minutes for real-time PCR. Furthermore, in this study, a real-time PCR instrument was employed for fluorescence signal detection and RPA products were diluted prior to their introduction into the CRISPR reaction in laboratory environment. Currently, many compatible devices are available to seamlessly integrate these two stages [21–24]. It should be noted that there are cost-effective fluorescence readers available as viable alternatives for signal detection. All of these advantages of this RPA-CRISPR/Cas12a combined method not only reduce equipment procurement and maintenance costs but also make this approach potential application for early diagnosis of *P. jirovecii* in primary healthcare settings. Future studies will be conducted to develop the two-stage integration process for the implementation of this method in primary healthcare facilities to effectively address this life-threatening infection.

One limitation of this study is the relatively small sample size (83 clinical specimens) which were obtained from a single center. This could have unintendedly introduced some degree of selection bias and so may not represent the broader population with different epidemiological patterns, which might limit the generalizability of the findings. In addition, the long-term performance and reliability of this CRISPR/Cas12a-based detection method was not assessed in this study. Future multi-center studies will validate the RPA-CRISPR/Cas12a assay’s clinical applicability by expanding its application to diverse respiratory patient cohorts and benchmarking against real-time PCR, thereby strengthening external validity. To further elucidate this diagnostic challenge, we are establishing a longitudinal cohort study incorporating host immune parameters and fungal load quantification, which will be reported in subsequent investigations. Addressing these limitations in future research will help to validate and potentially improve the clinical utility of this novel diagnostic approach. We point out that this is a qualitative detection method applied for screening only and so cannot distinguish colonization from infection, a vital clinical difference. Further quantitative analysis is still needed to assess its clinical significance, aiding in differentiating asymptomatic colonization from active infection for better clinical decision-making.

In conclusion, this study developed and validated a novel RPA-CRISPR/Cas12a-based detection method for *P. jirovecii* infection. Combined with the CRISPR/Cas12a detection system, the use of RPA for target DNA amplification, allows for not only rapid and reliable detection but also crucial timely clinical intervention. The high sensitivity and specificity making this new method a promising tool for early diagnosis of *P. jirovecii* pneumonia. Considering the limited sample pool from a single medical center, future research would be conducted with more comprehensive samples to further validate our findings. Additionally, studies are also necessary to streamline the workflow for point-of-care testing and evaluate the clinical utility and cost-effectiveness of this method in routine clinical practice. Overall, our study lays the groundwork for the potential implementation of CRISPR/Cas12a-based diagnostics in the management of *P. jirovecii* pneumonia. This approach serves as a potential tool for screening *P. jirovecii* pneumonia and suitability for deployment in primary healthcare facilities for this life-threatening infection.

## Supporting information

S1 Table crRNAs designed for detection *P. jirovecii.* (DOCX)

S2 Table Sequence of primers and crRNAs for the new assay. (DOCX)

S3 Table The real-time PCR and RPA-CRISPR/Cas12a detection results for 83 patient samples. (XLSX)

S4 Table Raw Data Supporting the article. (XLSX)
