# Supplementary material for: Establishment of an RPA-CRISPR/Cas12a combined diagnostic system for Pneumocystis jirovecii pneumonia
Source: PLoS Negl Trop Dis. 2025 Mar 18;19(3):e0012922. doi: 10.1371/journal.pntd.0012922 (PMC11918415; doi:10.1371/journal.pntd.0012922)
Supplement: S1 Table — (DOCX) [file pntd.0012922.s001.docx]

| **Table S1. crRNAs designed for detection *P. jirovecii*** | |
| --- | --- |
| Name | Sequences |
| crRNA5 | UAAUUUCUACUAAGUGUAGAUAAGUGGUGAACAGGUGAGUAAA |
| crRNA6 | UAAUUUCUACUAAGUGUAGAUUGAAUAGAUGAGUCUAAGUGG |
| crRNA7 | UAAUUUCUACUAAGUGUAGAUAGAGCCAAUGUGGUUAUUCAU |
| crRNA8 | UAAUUUCUACUAAGUGUAGAUAUUAGAUAACGUCAUAAUCCU |
| crRNA9 | UAAUUUCUACUAAGUGUAGAUUGUGUGAAGGAAUUAGUAUUC |
| crRNA10 | UAAUUUCUACUAAGUGUAGAUCAGCAUAAACUACAAGGGUAU |
| crRNA11 | UAAUUUCUACUAAGUGUAGAUAGUCUUGCGACAGUACUUCUC |
| crRNA12 | UAAUUUCUACUAAGUGUAGAUAUUCGAUAACCCACGAUAAAU |
| crRNA13 | UAAUUUCUACUAAGUGUAGAUACAACACGAACUAAAGACAGC |
| crRNA14 | UAAUUUCUACUAAGUGUAGAUCGUUCGUUAGCGGAAUAAACC |
