# Supplementary material for: Establishment of an RPA-CRISPR/Cas12a combined diagnostic system for Pneumocystis jirovecii pneumonia
Source: PLoS Negl Trop Dis. 2025 Mar 18;19(3):e0012922. doi: 10.1371/journal.pntd.0012922 (PMC11918415; doi:10.1371/journal.pntd.0012922)
Supplement: S2 Table — (DOCX) [file pntd.0012922.s002.docx]

| **Table S2. Sequence of primers and crRNAs for the new assay** | | | | |
| --- | --- | --- | --- | --- |
| Target gene | Primer | Sequences | Amplicon size | GeneBank accession No. |
| Mitochondrial small  subunit ribosomal RNA | PJ-RPA-7F | 5’-GGTGAACAGGTGAGTAAAGATAGAAATC-3’ | 159bp | JX499143 |
|  | PJ-RPA-7R | 5’-TCATTCTTTCAAATATAACTAGGGTTCTTAGG-3’ |  |  |
|  | crRNA5 | 5’-UAAUUUCUACUAAGUGUAGAU  AAGUGGUGAACAGGUGAGUAAA-3’ | **-** |  |
|  | crRNA6 | 5’-UAAUUUCUACUAAGUGUAGAU  UGAAUAGAUGAGUCUAAGUGG-3’ | **-** |  |
